# The 10/66 dementia research group - 10 years on

**Published:** 2009-01

**Authors:** Martin J. Prince

**Affiliations:** Department of Epidemiological Psychiatry, Centre for Public Mental Health, Institute of Psychiatry, King’s College, London

**Keywords:** Dementia research, developing countries, epidemiology of dementia, dementia diagnosis, dementia care

## Abstract

Well-designed epidemiological research is relatively lacking in low and middle income countries where two-thirds of the world’s estimated 24 million people with dementia live. The 10/66 Dementia Research Group has sought since 1998 to redress this imbalance. Pilot studies to develop and validate dementia diagnostic measures and study care arrangements in 26 centers worldwide were followed by one phase cross-sectional catchment area surveys in eight Latin American countries, China, India, Nigeria and South Africa. The protocol includes assessment of sociodemographics, disability, care arrangements, physical and mental health, and dementia diagnosis with (more restrictive) DSM-IV and (less restrictive) 10/66 dementia criteria. An incidence phase is underway in eight countries. 10/66 dementia prevalence is generally double that of DSM-IV dementia. DSM-IV dementia is particularly rare in India, attributable to the small proportion of family informants confirming cognitive decline and social impairment. Carer psychological and economic strain is as high as in the developed world, despite traditional family care arrangements. A significant minority of people with dementia are vulnerable due to lack of family support and economic resources. Earlier studies probably underestimated dementia prevalence in regions with very low awareness of this emerging public health problem. More research is needed to delineate the impact of dementia relative to other chronic diseases, and secular trends in countries experiencing rapid demographic ageing and health transition. Packages of care are also a priority - healthcare services and governments have not responded to families’ complex needs for support in their long-term care role.

## INTRODUCTION

The 10/66 Dementia Research Group (10/66) was founded ten years ago at the annual conference of Alzheimer’s Disease International (ADI) in Cochin, India. 10/66 refers to the less than one-tenth of all population-based research into dementia that had been directed towards the two-thirds or more of people with dementia living in developing countries. 10/66 was formed to redress this imbalance, encouraging active research collaboration between centers in different developing countries and between developed and developing countries. The Cochin symposium established priorities for 10/66, described in a consensus publication co-authored by the founding members.[1] More research was needed to describe prevalence and incidence and to explore regional variations using harmonized methods. Another priority was the description of care arrangements for people with dementia, quantifying the impact upon caregivers of providing care and evaluating the effectiveness of new services for people with dementia and their caregivers. The group identified potential through good quality research for generating awareness, pioneering service development and influencing policy. Now is an opportune time to review progress in India and in other regions. This review will focus, but not exclusively, upon the work of the 10/66 Dementia Research Group.

## FORMATIVE STAGE (1998-2003)

Methodological issues needed first to be addressed.[2] Accordingly, 10/66 carried out pilot investigations in 26 centers from 16 developing countries in Latin America and the Caribbean, Africa, India, Russia, China and SE Asia. 2885 persons aged 60 and over were interviewed, 729 people with dementia, and 3 groups free of dementia: 702 with depression, 694 with high education and 760 with low education. Seven centers in India participated in this phase of the project, two from Chennai, and one each from Bangalore, Goa, Hyderabad, Thrissur and Vellore. We aimed 1) to develop and validate a one-phase education- and culture-fair dementia diagnostic algorithm to be used in populations with very little formal education and in very different countries and cultures and 2) to obtain preliminary information on care arrangements for people with dementia, the impact upon caregivers of providing care and the common behavior problems that they encounter across regions and cultures.

### Dementia diagnosis

Our pilot studies demonstrated the feasibility and validity of a one phase culture and education-fair diagnostic protocol for population-based research.[3,4] The Geriatric Mental State (a structured clinical interview assessing dementia, depression and psychosis syndromes), the Community Screening Instrument for Dementia (cognitive test, and informant interview) and the modified CERAD 10 word list-learning task each independently predicted dementia diagnosis. A diagnostic algorithm derived in one half of the sample from all four elements performed better than any of them individually. Applied to the other half of the sample, it identified 94% of dementia cases with false positive rates of 15%, 3% and 6% in the depression, high education and low education groups. We concluded that 10/66 Dementia Diagnosis was “education-fair,” that is the false positive rate among those with low levels of education was low, and “culture-fair,” that is that equivalent validity was established for a wide variety of countries, languages and cultures. It therefore provides a sound basis for dementia diagnosis in clinical and population-based research, supported by translations of its constituent measures into many languages (Hindi, Tamil, Malayalam, Konkani, Mandarin/ Cantonese, Russian, Spanish, and Portuguese) covering the majority of the peoples of the non-English speaking world. For the 10/66 population-based surveys, we also decided to apply the DSM-IV criterion for dementia, having extended the scope of the one-phase assessment to ensure that the necessary data were recorded. Use of this criterion has been associated with strikingly low prevalences of dementia in some previous LAMIC studies,[5,6] and the relative concurrent and predictive validity of the two diagnostic approaches needed to be evaluated.

### Care arrangements and caregiver strain

In the pilot study, we interviewed 706 persons with dementia and their caregivers[7–9] Most caregivers were women living with the person with dementia in extended family households. One-quarter to one-half of households included a child. Larger households were associated with lower caregiver strain, where the caregiver was co-resident. However, despite the traditional apparatus of family care, levels of caregiver strain were at least as high as in the developed world. Many had cutback on work to care and faced the additional expense of paid carers and health services. Families from the poorest countries were particularly likely to have used expensive private medical services and to be spending more than 10% of the per capita GNP on health care. We concluded that the high levels of family strain identified in this study feed into the cycle of disadvantage and should thus be a concern for policymakers in the developing world.

### Behavioral and psychiatric symptoms of dementia (BPSD)

At least one behavioral symptom of dementia (BSD) was reported in 70.9% and at least one psychiatric syndrome was exhibited by 49.5%of the 555 people with dementia included in this sub-study[10] Depression syndromes (43.8%) were most common, followed by anxiety neurosis (14.2%) and schizophreniform/paranoid psychosis (10.9%). More advanced dementia, poorer functioning and the presence of depression or anxiety were each associated with BSD. BSD and psychiatric syndromes (anxiety neurosis and schizophreniform/paranoid psychosis) independently predicted caregiver strain after controlling for cognitive impairment. BPSD are poorly understood, leading to shame and blame. They may be taken by outsiders as prima facie evidence of neglect or abuse. Caregivers then face a double jeopardy: the strain of care heightened by the stigma and blame that attaches to them because of the disturbed behavior of their relative. We concluded that BPSD were common among people with dementia in developing countries. Representative population studies were needed to clarify prevalence and impact, but our research suggested considerable unmet need, with much scope for intervention.

### Awareness

Knowledge, attitudes and beliefs are best assessed through qualitative research studies. Three studies from India[11–13] (two conducted by the 10/66 Dementia Research Group) used a mixture of focus group discussion and open-ended interviews to investigate these issues. They tended to agree regarding the extent of awareness in the different communities studied. First, the typical features of dementia are widely recognized, and indeed named “Chinnan” (literally childishness) in Malayalam language in Kerala,[13] “nerva frakese” (tired brain) in Konkani language in Goa,[12] and “weak brain” in Hindi in Banares.[11] However, in none of these settings was there any awareness of dementia as an organic brain syndrome or indeed as any kind of medical condition. Rather, it was perceived as a normal, anticipated part of ageing. In Goa, the likely causes were cited as “neglect by family members, abuse, tension and lack of love.”[12] In Kerala, it was reported that most caregivers tended to misinterpret symptoms of the disease and to designate these as deliberate misbehavior by the person with dementia.[13] This general lack of awareness has important consequences. First, there is no structured training on the recognition and management of dementia at any level of the health service. Second, in the absence of understanding regarding its origins, dementia is stigmatized: for example, sufferers are specifically excluded from residential care and often denied admission to hospital facilities.[12] Third, there is no constituency to place pressure on the government or policy makers to start to provide more responsive dementia care services.[13] Fourth, while families are the main caregivers, they must do so with little or no support or understanding from other individuals or agencies. A critical mass of informed caregivers can assist awareness-raising, provide advice and support to families, and can work with Alzheimer associations to lobby for more services that better meet their needs. Community solidarity can effect change through support for policies based on equity and justice - a fairer distribution of healthcare services, and access to effective care regardless of age. Aware communities can provide support or at least not stigmatize and exclude those with dementia and those who care for them. Policymakers read newspapers and can be held to account by media campaigns backed up by advocacy from committed NGOs. In developed countries, dementia awareness is growing rapidly with the media playing an important part; coverage over18 months in the UK Daily Telegraph has increased from 57 articles in 1998/9 (1) to 112 in 2006/7. Recent evidence-based reports from the UK and the Australian Alzheimer associations garnered considerable media attention and were instrumental in making dementia a national health priority in both countries. Public awareness in LAMIC is less developed, with few media outlets carrying stories about dementia and ageing - a search in 1999 of the Times of India identified no articles (1). 10/66 research teams in Argentina, Venezuela, Peru, Dominican Republic and India have succeeded in getting the message out in newspapers, TV and radio. The Times of India published 15 articles in the last 18 months alone. Our experience is that while LAMIC media are receptive to these stories as part of their role in informing the public and stimulating debate, efforts are required to alert them to the importance of ageing and dementia and to build their capacity to report research and understand its local relevance.

## PREVALENCE PHASE (2003-PRESENT)

The evidence base on the prevalence of dementia was patchy in many world regions, hampering previous estimates of the global burden of the disease.[14,15] The 10/66 Dementia Research Group has now completed population-based surveys (2003-2007) of dementia prevalence and impact in 12 sites in eight low and middle income countries (India, China, Cuba, Dominican Republic, Brazil, Venezuela, Mexico, and Peru).[16–21] Further surveys are underway in Argentina, Puerto Rico, Nigeria and South Africa. Cross-sectional comprehensive one phase surveys have been conducted of all residents aged 65 and over of geographically defined catchment areas in each site with a sample size of 2000 in each country. The net result will be a unique resource of directly comparable data on over 20,000 older adults from three continents. All studies use the same cross-culturally validated assessments (dementia diagnosis and subtypes, other mental and physical health, anthropometry, demographics, extensive non-communicable disease risk factor questionnaires, disability/functioning, health service utilization, care arrangements and caregiver strain). A publicly accessible data archive has been established as a resource for the academic community (www.alz.co.uk/1066).

### The prevalence of dementia

The prevalence of DSM-IV dementia varied widely, from less than one percent in the least developed sites (India and rural Peru) to 6.4% in Cuba.[17] 10/66 dementia prevalence was higher than that of DSM-IV dementia and more consistent across sites, varying between 5.6% and 11.7%.[17] The discrepancy was explained by the observation that informants in the least developed sites, particularly India, were less likely to report cognitive decline and social impairment (an essential criterion for DSM-IV dementia diagnosis) even in the presence of objective memory impairment. After standardizing for age and sex, DSM-IV prevalence was similar in the urban Latin American sites to that in Europe, but in China the prevalence was only one half and in India and rural Latin America, one-quarter or less of the European prevalence. We concluded that the DSM-IV dementia criterion may underestimate dementia prevalence, particularly in regions with low awareness of this emerging public health problem. The higher 10/66 dementia prevalences were more consistent with a recent ‘Global Prevalence of Dementia’ expert consensus.[15]

The findings from the Indian 10/66 prevalence studies are compared with those of previous studies of the prevalence of dementia in India in [Table 1]. There are several points to be noted

**Table 1:** Studies of the prevalence of dementia in India India and China

Studies - setting (reference)	Design, sample size	Outcome	Age-specific prevalence (%) with 95% confidence intervals							Overall prevalence (age range)
			60-64	65-69	70-74	75-79	80-84	85-89	90+	
1. Ernakulam (rural)[25]	Three phase N = 2067	DSM-IIIR	0.33 0-0.79	0.99 0.55-1.43	1.50 0.3-2.7	3.24 1.16-5.32	12.88 7.78-17.98	16.28 8.48-24.08	32.14 14.86-49.42	3.19 (60+)*
2. Thiroporur (semi-rural)[26]	Two phase N = 750	ICD-10	2.5 1.25-3.75		5.5 1.5-9.5			16.0 1.6-30.4		3.5 (60+) 2.2-4.8
3. Ballabgarh (rural)[6]	Three phase N = 5126	DSM-IV CDR>=0.5		0.70 0.38-1.18		1.68 0.81-3.10		9.85 5.24-16.84		0.84 (55+) 0.61-1.13
										1.36 (65+) 0.96-1.88
4. Mumbai (urban)[22]	Three phase N = 24488	DSM-IV	0.28 0.05-0.51	0.80 0.38-1.22	2.42 1.54-3.30	4.99 3.04-6.94	5.06 2.72-7.40	3.85 1.24-6.46		2.31(65+) 1.84-2.78
5. Ernakulam constituency, Kochi (urban)[24]	Three phase N = 1934	DSM-IV	-	0.66 0.29-1.53	2.04 1.18-3.54	5.22 3.73-8.10	7.14 4.16-12.07	11.86 5.94-22.57	13.33 4.05-38.35	3.36 (65+) 2.73-4.07
6. Kolkata Municipal Corporation (urban)[23]	Three phase N = 5430	DSM-IV Men	0.38 0.18-0.82		0.79 0.39-1.63			2.27 1.12-4.60		1.02 (60+) 0.78-1.32
		DSM-IV Women	0.32 0.14-0.75		0.78 0.36-1.68			3.04 1.68-5.50		
7. Chennai (urban)[17]	One phase N = 1005	DSM-IV	M	0.6 0.0-1.7	1.6 0.0-3.8	No cases		No cases		0.9 (65+) 0.3-1.5
			F	0.8 0.0-2.0	1.1 0.0-2.5	No cases		3.0 0.0-7.3		
		10/66 Dementia	M	2.9 0.4-5.4	5.5 1.5-9.6	4.5 0.6-9.6		25.0 12.8-37.2		7.5 (65+) 5.8-9.1
			F	5.5 2.5-8.4	7.4 3.6-11.2	8.0 1.7-14.3		21.2 11.0-31.4		
8. Vellore (rural)[17,21]	One phase N = 999	DSM-IV	M	0.7 0.0-2.1	1.3 0.0-3.1	No cases		1.4 0.0-4.1		0.8(65+) 0.2-1.3
			F	0.5 0.0-1.6	1.0 0.0-2.4	No cases		1.5 0.0-4.4		
		10/66 Dementia	M	4.3 0.9-7.7	5.8 2.1-9.6	5.7 0.7-10.6		11.0 3.6-18.3		10.6 (65+) 8.6-12.6
			F	7.8 4.0-11.6	14.8 9.8-19.8	15.7 8.0-23.5		29.4 18.3-40.5		

Note: Confidence Intervals for studies 1, 2, 4, 5 and 6 were calculated from numerator and denominator data provided in the papers, with no account taken of the two or three phase design and the multi-stage sampling. This will have led to underestimation of the standard error, i.e., the robustly estimated confidence intervals would be wider.

1. The evidence base has expanded considerably since 1998, with over 40,000 older Indians now having been studied in eight centers, four predominately urban: Mumbai,[22] Kolkata,[23] Kochi[24] and Chennai;[17] and four predominately rural: Ballabgarh,[6] Ernakulam (Kerala),[25] Thiroporur (Tamil Nadu),[26] and Vellore (Tamil Nadu).[17–21]
2. The 10/66 studies in Chennai (*n* = 1005) and Vellore (*n* = 999) are small in size compared with most others, and cover only urban and rural districts in one state, Tamil Nadu.
3. The other studies all have relatively complex designs, comprising multistage (cluster) sampling and two or three phase dementia diagnosis (screening followed by definitive clinical dementia diagnosis on screen positives and a random sample of screen negatives). The 10/66 studies used a much simpler approach with whole catchment area sampling and one phase dementia diagnosis. While multistage sampling permits prevalence estimates to be generated for much larger base populations (for example, the whole population of Kolkata) sample weights need to be used to calculate prevalence and confidence intervals, and it was not clear that this had been done for several studies. One phase diagnosis offers several intrinsic advantages over two or more phases[27](study of a wider range of outcomes, no attrition between phases and increased efficiency when the sum of the sensitivity and specificity of the screening instrument is < 1.6). A bigger concern is that for several of the two or three phase studies in India, the method seemed not to have been applied in the recommended fashion.[28] Either screen negatives were not selected for the second phase[22] or weighting back was not carried out properly[25] or not carried out at all.[6] The net result is, inevitably, an underestimate of true prevalence and an overestimate of precision.
4. Notwithstanding these methodological concerns, the estimates of DSM-IV dementia prevalence in the 10/66 surveys were consistent with, or if anything a little lower than those of previous Indian studies. This may have been because DSM-IV diagnoses were applied strictly using a computerized algorithm, rather than through clinician judgment or consensus.[29]
5. The striking finding is the disparity between the prevalence of the cross-culturally validated 10/66 dementia diagnosis and that of DSM-IV or ICD-10 dementia in 10/66 and most other centers. Were the 10/66 dementia prevalences to be correct and generalizable, then we have calculated that our current best estimates of numbers of people with dementia in India[15] would need to be inflated by between two and a half and threefold.[17]

There are several possible explanations for the discrepancy between objective cognitive impairment and informant reports, noted in other less developed 10/66 centers as well as in India.[17] First, our cognitive tests may be biased, overestimating cognitive impairment in these settings. Second, objective cognitive impairment may be less likely to lead to noticeable impairment in the performance of normal social roles because of the high levels of instrumental support routinely provided to all older people, particularly in the early stages of dementia. More attention may need to be given to developing culturally relevant assessments to detect the consequences of early intellectual decline. Third, impairment/ decline may have been noted by informants, but they may have been reluctant to disclose this because of the culture of respect towards older people (supported by our finding of lower informant report scores for heads of household and male participants.[17] Fourth and finally, low awareness; impairment/ decline may have been noted, but attributed to “normal ageing”[12,13] and hence not worthy of mention given the implicit focus of the assessments upon abnormality. Our confidence in the validity of the 10/66 dementia diagnosis has been bolstered a) by the demonstration, in Cuba, that it agreed better with Cuban clinician diagnoses than did the DSM-IV computerized algorithm, which missed many recent onset and mild cases[29] and b) by the finding that levels of disability were similar for 10/66 dementia cases regardless of whether they were confirmed as cases by the DSM-IV dementia algorithm.[17] The scope for potential underdiagnosis is apparent and acknowledged in other Indian studies. In Kochi, Shaji[24] noted that while 55 people met DSM-IV criteria, a further 41 had cognitive impairment and functional impairment, and a further 127 cognitive impairment but no history of functional impairment. We would agree with his view that predictive validity may be the key to this conundrum. The essential feature of the dementia syndrome is that it is a progressive neurodegenerative disorder. We will be assessing this in our Chennai centre (and all other 10/66 centers), following up all DSM-IV, 10/66 dementia and “cognitive impairment no dementia” cases two to three years after baseline.[16] True cases will have progressed in cognitive impairment, functional disability and needs for care.

### Impact of dementia

Our first objective has been to provide a descriptive evidence-base on the prevalence and impact of dementia. Worldwide, surprisingly few epidemiological studies of dementia have gone beyond reporting on prevalence, incidence and etiology. The impact of dementia upon the individual, the family and the society has been little studied, particularly its contribution to disability, dependency, caregiver strain and costs. The response of health services and systems has also been relatively neglected. A preliminary comparative analysis of the circumstances of those with dementia in each centre, carried out for this review [Table 2] highlights some of the vulnerabilities of dependent older people living in these regions. Social protection is hard to define, depending on an interaction between health status and dependency on the one hand, income sufficiency and secure living arrangements. In the Dominican Republic, rural Peru and Mexico, rural China and in India, pension coverage is low and many people with dementia are significantly reliant on family cash transfers. In contrast with developed countries, it is relatively unusual for people with dementia to live alone or just with their spouse; living with children or children-in-law is the norm, and three generation households (including children under 16) were relatively common. Nevertheless, around one-fifth of people with dementia (10% to 37% by centre) were classified as having potentially vulnerable living circumstances. Many need long-term care, currently provided by family carers. Primary care services do not meet their needs. Governments neither provide long-term care nor support carers.

**Table 2:** Social protection for older people with dementia - 10/66 catchment area surveys in Latin America, India and China

	Income security					Secure living arrangements			Availability of family support		

Population- based centre catchment area	n	Receiving a government or occupational pension %	Receiving income from family transfers %	Receiving a disability pension %	Experiencing food insecurity %	Living alone %	Living with spouse only %	Total %	No children %	No children within 50 miles %	Total %
Cuba (urban)	323	81.4	7.4	0.9	5.6	6.3	10.2	16.5	16.5	3.0	19.5
Dominican Republic (urban)	242	27.3	23.6	0.8	13.7	8.5	10.2	18.7	12.0	13.1	25.1
Venezuela (urban)	146	41.1	2.7	4.1	2.7	5.7	4.9	10.6	7.8	5.6	13.4
Peru (urban)	130	58.5	5.4	1.1	1.6	1.6	9.4	11.0	16.4	0.0	16.4
Peru (rural)	36	66.7	0.0	0.0	8.6	13.9	8.3	22.2	19.4	5.7	25.1
Mexico (urban)	93	78.5	7.5	1.1	3.2	14.0	9.3	23.3	4.3	0.0	4.3
Mexico (rural)	87	34.5	17.2	2.3	12.6	16.5	11.1	27.6	4.6	1.2	5.8
China (urban)	84	84.5	11.9	0.0	0.0	2.5	34.5	37.0	0.0	0.0	0.0
China (rural)	56	10.7	23.2	0.0	3.6	3.6	8.9	12.5	3.6	4.2	7.8
India (urban)	75	13.3	28.0	2.7	28.0	4.0	13.3	17.3	5.3	0.0	5.3
India (rural)	108	26.9	44.4	0.0	17.6	15.1	5.7	20.8	8.3	2.6	10.9

### Other chronic diseases

In addition to dementia, the 10/66 one phase surveys include ascertainment of depression, other mental disorders, hypertension, dyslipidemia, diabetes, metabolic syndrome, heart disease, stroke, chronic obstructive pulmonary disease and arthritis[16] As well as providing a rich baseline for studying the etiology of dementia in the incidence phase, this will allow exploration of the relative, independent and interactive contributions of these various chronic conditions to disability, dependency, service utilization, economic costs and mortality. Despite the growing interest in chronic diseases in LAMIC[30,31] there are very little detailed data available on their prevalence and impact, with most studies focusing exclusively or mainly on young and middle-aged adults[32–34] The 10/66 Dementia Research Group studies constitute a unique opportunity to begin to chart the epidemiologic transition and its impact upon older persons.

With the first wave of prevalence studies complete, a series of papers on these topics is in preparation, and a schedule can be accessed on our project website (www.alz.co.uk/1066) together with links to abstracts of published works. Current priorities include

1. The contribution of dementia and other chronic diseases to disability and dependency
2. Carer strain and its correlates among carers of people with dementia
3. A descriptive study of care arrangements for people with dementia
4. The contribution of dementia and other chronic diseases to carer strain
5. Use of health services by people with dementia
6. Predictor of health service use.

Evidence from population-based research on ageing and dementia from low and middle income countries should help to stimulate a wider debate about older people’s health and social care needs, and how they should be met.[35]

## INCIDENCE PHASE (2007-2010)

The 10/66 Dementia Research Group has just begun a 2.5 to three year incidence phase follow-up of the baseline sample. The incidence phase will exploit the rich baseline cross-sectional data in the six Latin American countries (Cuba, Dominican Republic, Venezuela, Mexico, Peru and Argentina) and in China to assess risk factors for incident dementia, stroke and mortality. Verbal autopsy will be used to identify causes of death. The incidence phase will involve approximately 15,000 older people. First results should be available in early 2009, and the incidence phase will be completed in 2010. We will estimate the annual incidence rate of dementia and its subtypes, by age group, education and centre, and investigate risk factors for incident dementia and AD, focusing upon cardiovascular risk factors, micronutrient deficiencies and other dietary deficiencies, anemia and sub-clinical hypothyroidism. We will also seek to confirm the predictive validity of the survey dementia diagnoses (DSM IV and “the 10/66 dementia algorithm”) and Mild Cognitive Impairment (MCI) through three-year follow-up of all dementia and MCI cases, and to carry out a longitudinal study of evolving care arrangements for people with dementia, and caregiver strain.

## SERVICE DEVELOPMENT AND EVALUATION (ONGOING)

People with dementia and their families are particularly unlikely to access healthcare, despite the high levels of associated disability and caregiver strain. Lack of demand for services arises in part from a tendency to view dementia as a normal part of ageing. Encouraging help-seeking requires community dissemination to increase awareness with information from government, healthcare providers and media. However, efforts to increase demand must be accompanied by health system and service reform, so that help-seeking is met with a supply of better-prepared, more responsive services. In parallel with its epidemiological surveys, 10/66 has been testing the effectiveness of training community healthcare workers to identify people with dementia[36–38] and to deliver a brief intervention to educate and train caregivers.[16] Initial findings, from the first two of several randomized controlled trials in Goa[39] and in Moscow[40] show highly promising results. In practice, such interventions will need to be incorporated into horizontally constructed programs addressing the generic needs of frail, dependent older people and their caregivers, whether arising from cognitive, mental or physical disorders.

## FUTURE PRIORITIES

Clearly progress has been made. A recent Lancet review[41] highlights both the growing awareness of the importance of dementia in developing countries and the burgeoning evidence base. Nevertheless, much more research is needed to close the 10/66 gap. There is still a need for a definitive multi-centric prevalence survey in India with true nationwide scope, encompassing regional, cultural, ethnic, religious, socioeconomic and rural/ urban diversity. Such a survey, whether or not it used the 10/66 methodology, would be enhanced by a common protocol in all centers, with a one phase design including the ascertainment of comorbid chronic diseases, disability, needs for care, care arrangements and health service utilization. It would provide a much needed baseline to monitor, in future surveys, trends in demographic ageing and the health transition. Also missing, for India, is any large-scale prospective study of risk factors for dementia, with biological samples (DNA, hematology, fasting glucose and lipids and frozen serum for later evaluation), other cardiovascular risk factor exposures, diet and anthropometry all collected at baseline. The 10/66 Dementia Research Group has succeeded in establishing such a study across our Latin American network and, to an extent in China, but not yet in India where the need is just as great. Finally, there is a great need, as indicated above, to develop packages and programs of care for people with dementia and their caregivers, which, their cost-effectiveness having been established, would be capable of being scaled up across India’s complex mixed healthcare system.
